# Evaluating Telemedical Supervision for Critical Anesthesia Scenarios: Randomized Controlled Simulation Study

**DOI:** 10.2196/88056

**Published:** 2026-08-18

**Authors:** Jonas Roth, Verena Voigt, Michael Schauwinhold, Andreas Follmann, Michael Czaplik

**Affiliations:** 1 Department of Anaesthesiology Medical Faculty Universitätsklinikum Aachen Aachen, North Rhine-Westphalia Germany

**Keywords:** telemedicine, telesupervision, interoperability, service-oriented device connectivity, SDC, anesthesia

## Abstract

**Background:**

Telemedicine may improve access to specialized care, but its use for supervision during critical anesthesia situations remains underexplored. A standardized tele-supervision (TSV) solution for operating rooms (ORs) is lacking.

**Objective:**

This study aimed to evaluate a novel telemedical supervision system for critical anesthetic scenarios in a simulated OR environment and compare it to traditional on-site supervision. Specifically, adherence to standard operating procedures (SOPs), the number and modality of senior physician contacts, workload, and user perceptions were assessed.

**Methods:**

In this randomized controlled simulation study, 16 anesthesiology residents in their first 2 years of training at the Uniklinik Rheinisch-Westfälische Technische Hochschule (RWTH) Aachen (Germany) were randomized using block randomization into 2 groups. The intervention group received remote support exclusively via a TSV system, while the control group used a conventional phone with on-site support. The telemedical system comprised an anesthesia workstation that integrated data from the patient monitor, anesthesia device, and syringe pumps using the Institute of Electrical and Electronics Engineers (IEEE) 11073 Service-Oriented Device Connectivity (SDC) standard and a mobile supervision workstation that enabled the senior physician to monitor multiple ORs and communicate via text, audio, and video. The simulated scenario involved a male patient aged 51 years undergoing an appendectomy who developed an anaphylactic reaction 3 minutes after receiving cefuroxime. Primary outcomes focused on the completion rate of necessary SOP measures. Secondary outcomes included workload, measured using the NASA Task Load Index (NASA-TLX), and participants’ postscenario questionnaire responses. Statistical comparisons were performed using a Welch *t* test.

**Results:**

All participants in both groups contacted the senior physician at least once. The control group had a mean of 6.44 (SD 4.80) SOP measures supported by the senior physician, whereas the intervention group had a mean of 5.00 (SD 3.06). The mean SOP completion rate was 92.5% (SD 0.04%) in the control group and 91.6% (SD 0.05%) in the intervention group, with no significant difference (*t*_11.94_=0.439; *P*=.67). NASA-TLX scores revealed that compared to the control group, there was lower mental demand in the TSV group but higher temporal demand. Subjective evaluations indicated mixed preferences regarding on-site support; however, most participants acknowledged the TSV system as a viable alternative when on-site support was not feasible.

**Conclusions:**

This study demonstrated no statistically significant differences between the groups, indicating similar performance with high adherence to SOPs and comparable clinical decision-making in a simulated high-stakes environment. Despite increased temporal workload, user feedback was positive, underscoring the system’s potential to address staffing shortages and resource limitations. Further research in real clinical settings is needed to optimize usability and validate these findings.

## Introduction

The rapid evolution of telemedicine has transformed health care delivery by improving access to specialized care, reducing costs, and enabling timely interventions in diverse clinical settings. Numerous systematic reviews and meta-analyses have demonstrated that telemedicine is effective in critical care, emergency medical services, and chronic disease management, where remote monitoring and consultation have enhanced patient outcomes or have been shown to be noninferior to conventional solutions [1-3]. In anesthesiology, telemedicine has been successfully applied to preoperative evaluations, intraoperative monitoring, and postoperative follow-up, thereby improving workflow efficiency and patient safety [4-8]. For example, tele-preoperative assessments have been associated with high patient satisfaction and comparable surgical outcomes compared to traditional in-person evaluations [9-11].

Despite these promising advances, a universal telemedicine solution tailored specifically to the complex environment of the operating room (OR) has yet to be established. In the OR, where rapid and precise decision-making is crucial, the integration of real-time data from multiple medical devices with effective remote supervision remains a significant challenge. Existing systems are often limited by technological readiness, inconsistent user training, and variability in clinical scenarios, highlighting the need for a standardized, robust telesupervision (TSV) platform for anesthesia care [12].

Because of these limitations, the development and adoption of an interoperable approach could offer a widespread and sustainable solution for TSV in the OR. The prototype used in this study leverages the Institute of Electrical and Electronics Engineers (IEEE) 11073 Service-Oriented Device Connectivity (SDC) interoperability standard [13,14], which enables seamless integration of critical patient and device data. Although this prototype was previously evaluated for general usability and user feedback in less critical scenarios, its performance in managing potentially life-threatening events within the OR has not been assessed. This study, therefore, aims to build upon prior work by evaluating the system’s effectiveness in a high-fidelity, simulation-based environment under conditions that mirror real clinical challenges. By comparing its performance to that of traditional on-site supervision in a potentially life-threatening scenario, we aim to demonstrate that remote support can maintain adherence to standard operating procedures (SOPs) without compromising clinical decision-making or increasing workload. The ultimate goal is to bridge the gap between the demonstrated strengths of telemedicine in other fields and the specific demands of anesthesia supervision in the OR.

## Methods

### Telemedical Supervision System

The telemedical supervision system used in this study was developed under the PriMed—Process Optimization Through Integrated Medical Devices in the Operating Room and Clinic research project, funded by the German State of North-Rhine Westphalia and the European Commission—European Regional Development Fund (Europäischer Fonds für regionale Entwicklung [EFRE]; grant EFRE-0801359) [15]. This system comprises 2 primary components: the anesthesia workstation located within the OR and a mobile supervision workstation for the senior physician.

The anesthesia workstation integrates data from the patient monitor, anesthesia machine, and syringe pumps within the OR using the IEEE 11073 SDC interoperability standard [13,14]. This integration facilitates the reception and display of data on the anesthesia workstation. Additionally, the anesthesia workstation provides communication options, allowing the anesthetist to connect with the senior physician via text chat or audio or video call.

On the senior physician’s side, the supervision workstation mirrors the data collected from the medical devices in the OR and offers the capability to switch between multiple ORs. It enables the supervisor to contact anesthetists in each OR using a telemedical module (“TeleDoc”; Docs in Clouds TeleCare GmbH). Once an audio or video connection is established, the supervisor can choose to view the anesthetist through the OR’s webcam or use a ceiling-mounted pan-tilt-zoom (PTZ) camera. This PTZ camera allows remote observation within the OR, enabling inspection of specific points of interest related either to the patient or to the overall scenario.

The supervision workstation has 3 key features.

The first feature is data integration and access. All critical patient and device data available on the anesthesia workstation through the SDC protocol are transmitted to the supervision workstation, enabling senior physicians to monitor multiple ORs from a single device. However, for this study, a proprietary protocol was used to transfer the data to the senior physician because of the lack of SDC capabilities of the devices used.

The second feature is communication capability. The telemedical module supports real-time text, audio, and video communication between the supervision workstation and each OR workstation, providing senior physicians with a reliable communication channel.

The third feature is mobile and lightweight design. The supervision workstation was designed as a fast, lightweight web application optimized for low-end mobile devices, such as tablets. Built with JavaScript and *React.js*, it is accessible through standard web browsers, ensuring portability and ease of use on various devices.

The integration of these workstations and the addition of the telemedical module created a functional, flexible system that enables senior anesthetists to support their colleagues remotely while maintaining critical access to real-time patient and device data.

### Study Design

Previous discussions about the system used and an evaluation of its acceptance by users highlighted its potential [15]. The goal was not to replace on-site senior physicians but instead to offer aid for hospitals and scenarios where currently no senior physician is present because of cost or staffing constraints. However, previous studies were limited to simple simulation scenarios that were rather uncritical for the resident anesthetist. This study focused on potentially life-threatening scenarios.

This randomized controlled evaluation study was designed to involve anesthesiology resident physicians from the Uniklinik Rheinisch-Westfälische Technische Hochschule (RWTH) Aachen (Germany) in their first 2 years of training. Due to shortages during the COVID-19 pandemic, the original goal of recruiting 20 participants was not reached; ultimately, 16 participants were recruited and randomly assigned to 2 groups using block randomization with 2 blocks of 10 participants each.

Given the early development stage of the supervision system, its evaluation was conducted using a human patient simulator (HPS; Elevate Healthcare) in a simulated OR. Anesthetists were confronted with a realistic anesthesiology scenario to assess the system’s effectiveness through a comparative approach.

In the defined scenario, for dealing with a critical event, the first group (intervention group) could receive support only via TSV, without being able to call the senior physician by phone and ask them to come to the OR. The second (control) group had access to a conventional phone for calling assistance and could ask the physician to be present in the OR.

To ensure a meaningful evaluation of the telemedical system’s capabilities, we deliberately chose not to allow the intervention group to summon the senior physician for in-person support. Although including this option might have further reinforced the comparability of outcomes—particularly if participants chose not to use it—we recognized a significant risk: participants might default to using the TSV system merely as a digital phone to request the supervisor’s physical presence. This concern was based on the participants’ habitual use of phone-based communication in clinical practice and their lack of prior exposure to TSV tools. By removing the fallback to in-person support, we sought to isolate and rigorously evaluate the functionality and effectiveness of remote supervision alone. This edge-case design enabled us to test whether performance under telemedical guidance, without any possibility of on-site intervention, could still match that under conventional supervision in a high-stakes clinical scenario.

To test the efficacy of the TSV system under this constrained setup, the following high-fidelity scenario was developed to simulate a critical medical emergency. The patient—a male aged 51 years weighing 85 kg and measuring 183 cm, with no known allergies—underwent an appendectomy. The surgeon requested the administration of cefuroxime as an antibiotic, to which the patient exhibited an anaphylactic reaction. Participants were required to react appropriately according to the SOPs of the Uniklinik RWTH Aachen, based on the “Guideline for Acute Therapy and Management of Anaphylaxis” [16]. This SOP was divided into basic measures applicable immediately after diagnosing a potential anaphylactic reaction and further measures necessary for different stages and severity levels of anaphylaxis (Multimedia Appendix 1).

The primary outcome measure was the proportion of correctly executed necessary SOP measures, calculated as the number of correctly performed measures divided by the total number of measures deemed necessary for each case. On the basis of the simulation model, early proactive reactions could prevent a more severe anaphylactic reaction, rendering certain measures unnecessary, while delayed or inadequate responses could lead to more severe reactions requiring additional interventions.

Observations also recorded whether these measures were executed at the participant’s own discretion or were instructed by the senior physician. Three minutes after the administration of cefuroxime, the HPS operator triggered the first stage of the anaphylactic reaction.

Secondary outcomes included workload, measured using the NASA Task Load Index (NASA-TLX) [17], and participants’ postscenario questionnaire responses, with different questions for the 2 groups. Although the intervention group was interviewed about its feedback and impression of the TSV system, the control group was asked whether it could imagine solving the situation with telemedical support and whether the presence of the senior physician was important to its members.

The NASA-TLX is a widely used tool to assess perceived workload, weighted from 0 to 100, across 6 dimensions: mental demand (the degree of cognitive effort required for the task), physical demand (the level of physical activity involved), temporal demand (the pressure related to time constraints), performance (self-assessed success in task execution), effort (the total mental and physical effort exerted), and frustration (the extent of stress, irritation, or satisfaction experienced during the task).

Video recordings from 3 cameras and screen recordings of the patient monitor were used for postscenario evaluations. Participants practiced the “thinking-aloud protocol” prior to the scenario, encouraging them to verbalize their actions and thoughts, thereby allowing precise monitoring of behavior and actions. The completion of corresponding SOP measures was logged, and each contact with the senior physician was noted, including the situation, reason, and time stamp.

Each simulation cycle involved an anesthesia nurse familiar with the simulation environment to support the anesthetist in the OR, a simulation technician from Aachener Interdisziplinäres Trainingszentrum für medizinische Ausbildung (AIXTRA), a technical manager to control the HPS and scenario settings, and a senior physician to address any questions from the participant.

### Ethical Considerations

All participants provided written informed consent before participating in the study. Participants were pseudonymized, and data protection and confidentiality were maintained in accordance with institutional protocols. Video recordings used to verify participants' actions, measures, and behavior were deleted after data acquisition. Participants did not receive any compensation for their participation. The study was approved by the Ethics Committee of the Uniklinik RWTH Aachen (EK 314-21). The participants were all medical residents who participated in a simulated scenario, so there were no direct health-related outcomes, and thus the study was not preregistered.

### Statistical Methods

For the comparison of the performances of the 2 groups, an independent 2-sample *t* test (Welch *t* test) was used. To check its applicability, a Shapiro-Wilk test was performed to test for normal distribution, and the Levene test was performed to assess the homogeneity of variances.

## Results

### Observation of Performance and Use

A total of 16 resident anesthetists from the Uniklinik RWTH Aachen, all in their first 2 years of postgraduate specialty training, were recruited and included in the study. Of these, 9 (56%) were female and 7 (43.8%) were male, with ages ranging from 25 to 35 years. Their clinical experience as anesthetists varied from 2 to 25 months. Participants were randomly assigned to either the control group (n=9, 56.2%) or the intervention group (n=7, 43.8%) using block randomization with a block size of 10 to ensure balanced group distribution.

During the simulated anesthesia scenario, in which a patient was at risk of developing anaphylactic shock, all resident anesthetists contacted the senior physician and requested support at least once.

The amount of assistance requested, defined as the number of SOP measures for anaphylactic shock in which the senior physician was involved, varied among participants. The proportion of supported SOP measures ranged from 0% to 79% in the control group, with a median of 39%. In the intervention group, this proportion ranged from 0% to 64%, with a median of 43%. Within the control group, the mean number of supported SOP measures was 6.44 (SD 4.80), whereas the intervention group had a mean of 5 (SD 3.06) supported measures.

According to the study setup, different communication methods were used in the 2 groups when senior assistance was required. In the intervention group, communication was exclusively remote via the tele supervision workstation. In contrast, participants in the control group primarily used the phone to request in-person support from the senior physician. In the control group, 79% (46/58) of the SOP measures assisted by the senior physician were carried out in person, while 12 (21%) were supported via phone communication.

The diagnosis of anaphylaxis was made independently by 4 (44%) of the 9 resident anesthetists in the control group and 4 (57%) of the 7 resident anesthetists in the intervention group.

Furthermore, the completion rate of necessary SOP measures was examined. The percentage of required measures performed varied depending on how early anaphylaxis was detected and treated, as some SOP steps for severe anaphylactic shock remained unnecessary when early interventions were successful. The mean completion rate was 92.5% (SD 0.04%; median 93.8%) in the control group and 91.6% (SD 0.05%; median 92.3%) in the intervention group. The IQR was 88.9% to 94.7% for the control group and 87.5% to 93.5% for the intervention group.

To test for a normal distribution, the Shapiro-Wilk test was performed, resulting in a *P* value of .27 for the control group and a *P* value of .33 for the intervention group. As both *P* values were >.05, we do not reject the null hypothesis that the data are normally distributed. To test for equality of variances, the Levene test was used and yielded *P*=.81. This high *P* value suggests that we do not reject the null hypothesis that the variances of the 2 groups are equal.

Thus, we were able to use the independent 2-sample *t* test (Welch *t* test), with t_11.94_=0.439, which showed no significant difference (*P*=.67) between the 2 groups.

### Evaluation of the System and Workload by Participants

**Figure 1:**
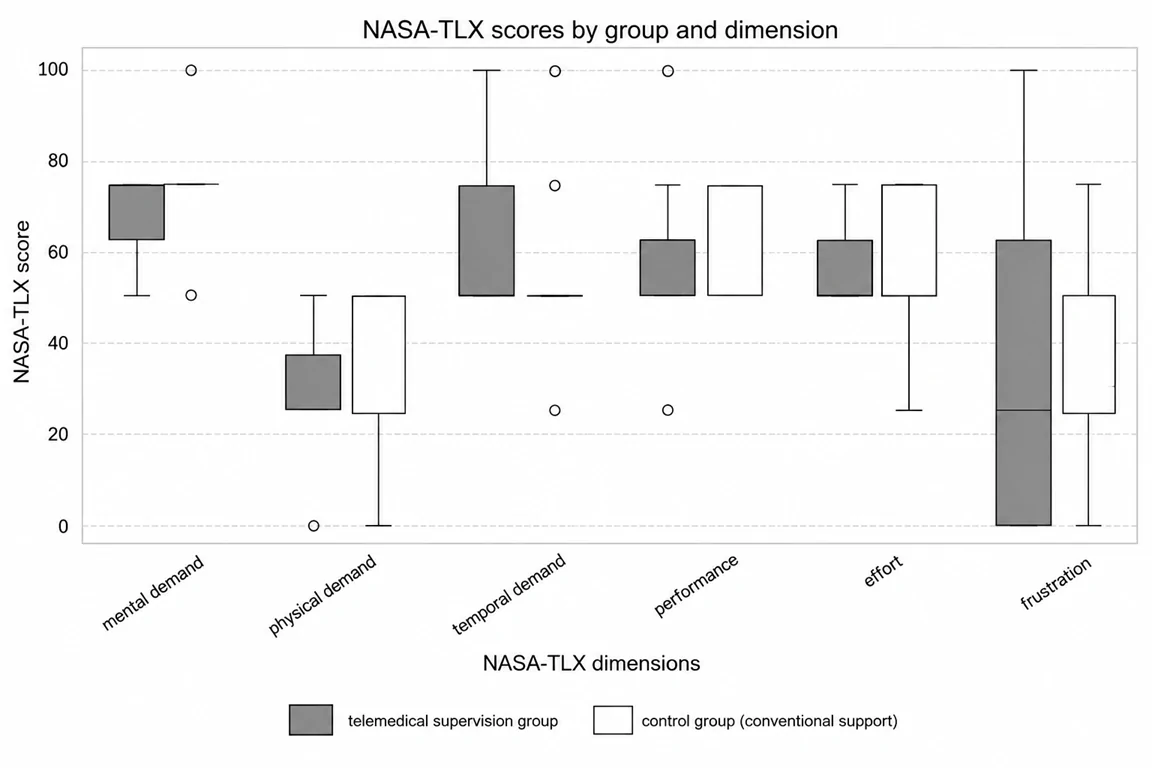
Assessment of perceived workload for both groups (white: control group; gray: intervention group) using the NASA Task Load Index (NASA-TLX), evaluating the demands across the 6 dimensions on a scale of 0 to 100.

After the simulation, participants completed the NASA-TLX questionnaire to assess workload (Figure 1). Mean scores and SDs for both groups are shown in Table 1.

**Table 1:** Mean scores for the 6 NASA Task Load Index (NASA-TLX) categories in the 2 groups.

NASA-TLX dimension	Intervention group, mean (SD)	Control group, mean (SD)
Mental demand	67.9 (12.2)	77.8 (15)
Physical demand	28.6 (17.3)	30.6 (16.7)
Temporal demand	64.3 (19.7)	55.6 (20.8)
Performance	57.1 (23.8)	58.3 (12.5)
Effort	57.1 (12.2)	55.6 (16.7)
Frustration	35.7 (40.5)	30.6 (24.3)

The TSV setup was rated as mentally and physically less demanding. However, temporal demand was rated differently, with the TSV setup being rated as more demanding. In all other workload categories, the perceived demand was very similar.

Participants in the intervention group were asked whether they would have preferred the option to call the senior physician on-site. Overall, 2 (28.6%) participants rather agreed, 3 (42.9%) were neutral, and 2 (28.6%) rather disagreed.

Participants in the control group were asked whether they believed they could have managed the scenario using the telemedical system and whether the presence of the senior physician was important to them. Of the 9 participants in the control group, 5 (55.6%) agreed or rather agreed that they could have handled the situation with the telemedical system, while the remaining 4 (44.4%) participants were neutral. Of the 9 participants in the control group, 7 (77.8%) considered the senior physician’s physical presence important, while 2 (22.2%) rather disagreed.

### Feedback on the TSV System

**Figure 2:**
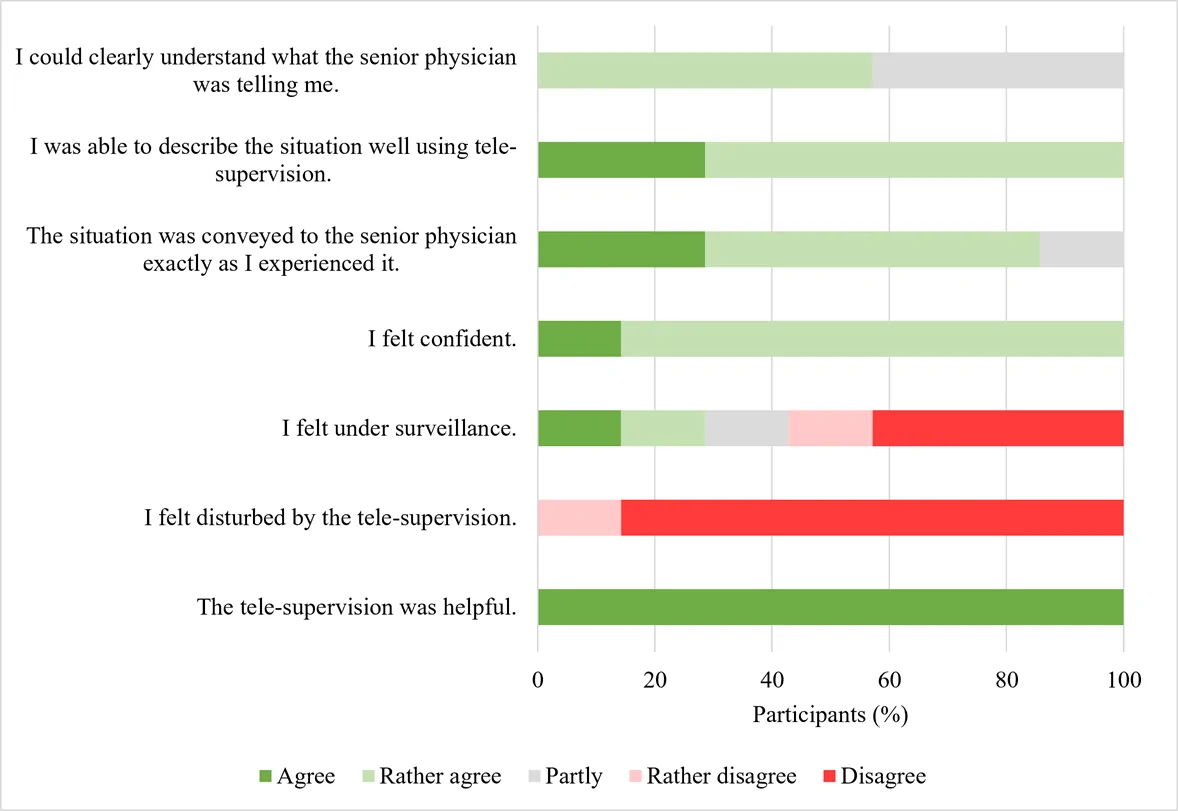
Feedback on the use of the telesupervision system by the intervention group, assessed through a questionnaire.

Participants who used the TSV system provided feedback on 7 predefined statements (Figure 2). None of the participants reported difficulties in understanding the senior physician or describing the situation effectively using the system. All participants perceived that the senior physician was able to assess the situation appropriately using the system. Additionally, all participants reported feeling confident during the scenario.

Responses regarding whether the system created a sense of being under surveillance were mixed. No participants reported feeling disturbed by the system, and all participants agreed that the system was helpful.

## Discussion

### Principal Findings

This randomized controlled simulation study investigated the effectiveness of a telemedical supervision system for anesthesiology residents managing a life-threatening intraoperative emergency. Participants were assigned to either a TSV group, supported remotely via a dedicated remote senior physician, or a control group with the conventional setup, in which a senior physician could be consulted by phone or requested for on-site support. The primary outcome was the completion rate of necessary SOP measures during an anaphylactic reaction triggered in a simulated patient. Both groups achieved similarly high SOP adherence, with no statistically significant difference between them, suggesting that remote supervision using the telemedical system did not compromise clinical performance in this high-fidelity simulation. These findings align with evidence from intensive care settings, where remote monitoring has maintained care quality and improved workflow efficiency [18]. Secondary findings revealed that although participants under TSV reported slightly lower mental demand, they experienced higher temporal demand. However, subjective evaluations highlighted general acceptance of the system, although some participants still preferred the physical presence of supervisors.

### Observation of Performance and Use

One of the key findings of this study is that both groups frequently sought assistance from senior physicians, with all participants reaching out at least once during the simulated anaphylaxis scenario. Given that all participating residents had less than 2 years of anesthesiology experience, this result emphasizes the critical importance of having a senior physician readily available for consultation in complex or deteriorating situations—both to ensure patient safety and to support clinical decision-making.

From a legal and organizational perspective, this is particularly relevant in Germany, where current regulations stipulate that a fully qualified anesthesiologist (“*Facharzt*”) must be immediately available when procedures are carried out by a resident. Traditionally, this has meant one-to-one supervision with physical presence or rapid on-site availability. From a legal and organizational perspective, these findings suggest that telemedical supervision may have the potential to contribute to fulfilling the requirement for immediate senior physician availability. However, this simulation study does not constitute legal validation, and whether TSV satisfies applicable regulatory requirements must ultimately be evaluated in real clinical practice and interpreted within the relevant legal framework. Compared to other previously proposed or used systems for telemedical support in anesthesia [6-8], the use of the IEEE 11073 SDC interoperability standard allows the full integration of secure data from connected medical devices. To date, telemedical solutions have either provided only a video signal or included selected parameters that were received proprietarily. Building upon a standardized interoperable protocol would enable widely applicable solutions, providing access to any captured or measured data points in a manufacturer-independent way and providing a foundation for more advanced analytical tools or decision support systems.

The study also observed that, in both groups, more than one-third of the executed SOP measures were completed with support from the senior physician. This further highlights the importance of expert supervision during the early training stages. Notably, in the control group, 79% (46/58) of all SOP measures assisted by the senior physician were carried out in person rather than via phone. This finding suggests that most critical assistance in conventional workflows still depends on physical presence. However, the comparable performance of the TSV group implies that the novel system could substitute many of these in-person support functions in this simulated scenario, providing remote clinical guidance without reducing SOP adherence.

In approximately half of all cases, the diagnosis of anaphylaxis was not made independently by the resident but required senior physician input, suggesting that, without guidance, outcomes could have been critical for the simulated patient.

Although the study focused on the German legal framework, it is important to note that in many other countries, particularly in resource-limited or rural settings, it is common for1 senior physician to supervise multiple ORs simultaneously—or even for nonphysician anesthesia providers to operate under remote supervision. In these settings, as seen in prior studies on tele-anesthesia and surgical tele-support [19], remote solutions have been key to enabling equitable care delivery. The demonstrated ability of TSV to maintain protocol adherence and decision-making quality could be particularly impactful. This approach may help standardize the quality of care across facilities and bridge geographic disparities in specialist availability.

Additionally, although this study exclusively assessed resident-initiated contact, it is conceivable that future implementations could include proactive monitoring by supervisors. A system like the one tested here would enable a senior physician to observe multiple ORs simultaneously and intervene early when needed, thereby providing a scalable and legally compliant alternative to conventional on-site oversight.

Finally, the completion rate of necessary SOP measures, which served as the primary objective parameter to monitor performance in this study, was nearly identical between the 2 groups. The control group achieved a mean SOP completion rate of 92.5%, while the intervention group achieved a mean rate of 91.6%.

These findings indicate that the TSV system did not compromise residents’ adherence to protocol during a critical incident.

An interesting observation was that 79% (46/58) of all senior physician–assisted SOP measures in the control group were ultimately supported in person rather than via telephone alone. This likely reflects a limitation of conventional phone-based communication, which provides only verbal information and often requires the supervisor to enter the OR to obtain a complete understanding of the situation. In contrast, the TSV system provided simultaneous access to patient data, medical device information, and audiovisual communication, potentially replacing many situations in which physical presence would otherwise have been necessary. Therefore, the observed preference for in-person support in the control group should not be interpreted as evidence that physical presence is inherently superior but rather as evidence that conventional telephone communication alone may be insufficient for many complex intraoperative situations.

### Evaluation of the System and Workload by Participants

However, the participants’ subjective experiences, as captured by the NASA-TLX, revealed some distinctions. Although the intervention group reported less mental demand when using the novel system, they perceived greater temporal demand. This finding is interesting, as it highlights the potential cognitive challenges associated with managing remote support provided through a technological solution during a high-stress situation. Being supervised remotely can heighten the feeling of being observed and evaluated without the personal rapport of in-person supervision, possibly making participants feel pressured to perform quickly. Conventional in-person supervision may represent a more familiar interaction, at least until such systems become more widely used and integrated into education and training.

When asked about their preferences, 2 (28.6%) of the 7 participants in the intervention group expressed a desire for on-site senior physician support, with 2 (28.6%) participants leaning toward agreement. In contrast, more than half of the control group indicated that they believed they could have managed the situation using the TSV system. Still, most valued the physical presence of the senior physician, with 7 (77.8%) of the 9 participants finding it important. Although this study focused on the experiences of the resident physicians, it must be noted that TSV may pose additional challenges for the supervising senior physician as well. Managing critical events remotely, without being physically present, can be disorienting, cognitively demanding, and may introduce a perceived or real sense of medicolegal risk. This study used only 1 experienced senior physician for all simulations, which limited the scope for evaluating this aspect. Future research should explore how TSV is perceived and handled by multiple supervising physicians across various levels of experience, to better understand its implications on both sides of the interaction.

Although some participants expressed a preference for the senior physician’s physical presence, this should not be generalized as a universal requirement. The need for on-site support may vary considerably depending on the nature of the problem being addressed. Organizational or procedural questions might be efficiently handled via phone, while more complex clinical issues may benefit from TSV, particularly when shared visual access to monitors and vital signs is crucial. Some scenarios, such as those requiring manual intervention, may truly demand physical presence in the OR. The size of this subset remains an open question. Future studies should aim to categorize the types of supervisory needs and identify their optimal support modalities. Additionally, the level of experience of the resident anesthetist plays a critical role. Although this study focused on early-stage residents with less than 2 years of experience, it is plausible that after approximately 12 months of practice, many clinical situations could be managed safely and effectively under remote supervision. Investigating the threshold of clinical experience required for safe and efficient TSV could inform staffing models that allow 1 senior physician to supervise multiple ORs without compromising patient safety. In addition, increasing staffing shortages and constrained resources in rural areas might be important limiting factors [20,21], for which TSV could provide a solution.

### Feedback on the TSV System

Regarding the user experience of this first demonstrator, participants using the system generally provided positive feedback regarding its functionality. They reported no issues in communicating with the senior physician and felt confident throughout the scenario. However, a notable proportion of participants expressed mixed feelings regarding the sensation of being under surveillance, even though none felt disturbed by the system. Indicators on the system interface or an indicator light on the webcam could potentially mitigate concerns about being observed without notice; however, this possibility was not further investigated in this study. Despite this, all participants agreed that the system was helpful during the scenario.

### Limitations

The study was conducted in a patient simulator environment, which, although highly controlled, might not fully replicate the complexities of real-world clinical situations. This could affect the generalizability of the findings. A similar demonstrator with a higher technology readiness level should be further evaluated in a real clinical environment with a safe fallback option.

Another key limitation is the small sample size and the exclusive focus on a single type of critical incident (anaphylaxis). The relatively small sample size limits the ability to detect small differences between groups. Although no statistically significant differences were observed for the primary end point, the study should be regarded as exploratory and was not powered to establish equivalence or noninferiority. Larger studies will be required to confirm these findings and to evaluate secondary outcomes with greater statistical precision. Broader scenarios involving various intraoperative emergencies, as well as a more diverse participant pool, will be necessary to generalize the results across different clinical contexts. The selection of a single anaphylaxis scenario was intended to provide a standardized and reproducible evaluation of the TSV system. Future studies should investigate additional intraoperative emergencies, such as major hemorrhage, difficult airway situations, or cardiac arrest, to assess the generalizability of the findings across a broader spectrum of clinical challenges. Moreover, the current evaluation focused solely on resident physicians with <2 years of clinical experience, without stratifying results based on experience level. Resident experience ranged from 2 to 25 months and was not used as a stratification variable during randomization. Although all participants were within the first 2 years of anesthesiology training, differences in experience may have influenced diagnostic accuracy, help-seeking behavior, and workload perception. Future studies with larger cohorts should stratify participants by training level or include experience as a covariate in the statistical analysis.

Although the completion rate of SOP measures was used as the primary outcome, the timing of individual actions was not systematically analyzed. In acute anesthetic emergencies, time to diagnosis and time to treatment may be clinically just as important as the eventual completion of SOP measures. TSV has the theoretical advantage of enabling immediate expert involvement without the delay associated with physically traveling to the OR. However, because both groups in this simulation received immediate access to senior physician support once contact was initiated, the present study was not designed to evaluate potential time-related benefits. Future studies should include time-to-event metrics, such as time to diagnosis, time to first epinephrine administration, or time to completion of critical interventions, to better assess potential efficiency gains associated with TSV.

A further limitation concerns the 1-sided perspective of the feedback: this study collected only residents’ impressions. Feedback from senior physicians, who supervised all simulations remotely, would provide valuable insight into usability, cognitive workload, clinical safety, situational awareness, and potential medicolegal concerns from the supervisor’s perspective. However, only 1 senior physician participated in this study, precluding such analysis. Including multiple senior physicians in future research will be essential to assess interindividual variation, supervisor acceptance, and broader applicability.

Another design constraint was that the TSV group was not permitted to use a phone or request the supervisor’s physical presence. Although this design enabled a clearer evaluation of the stand-alone telemedical system, it does not reflect the real-world flexibility of hybrid models (eg, initial tele-consultation followed by physical support if needed). Future studies could allow participants to freely choose between telemedical support, phone calls, and summoning the supervisor on-site. However, this would require significantly larger sample sizes and more complex study protocols to account for the resulting variability. At the same time, other potential benefits of widespread implementation of this novel telemedical supervision system were not directly evaluated either. The system used in this study would allow the supervisor to seamlessly switch between different ORs and potentially even between different clinics. This could offer a huge potential for process optimization and more efficient use of personnel resources.

Finally, the TSV system evaluated in this study was still in the prototype stage. The user interface (UI) was in the early stages of development, and full integration of device data and clinical information had not yet been achieved. Future iterations could benefit from enhanced UI design, more intuitive interaction mechanisms, and real-time integration of patient data streams, which may further increase effectiveness, usability, and user acceptance.

### Conclusions and Outlook

In this randomized controlled study, we evaluated a telemedical supervision system for anesthesia residents managing critical scenarios. The study demonstrated performance similar to traditional on-site supervision, with similar completion rates of necessary SOP measures. Despite increased temporal demands reported by participants, the system was well received overall, highlighting its potential to address staffing shortages and resource limitations.

These findings carry important implications for the future of telemedicine in anesthesiology and beyond. First, the comparable adherence to SOPs in a high-stakes scenario suggests that TSV could be a viable option in situations where on-site senior staff is unavailable. This may be particularly relevant for rural hospitals, understaffed departments, or nighttime operations, where immediate in-person support may not always be feasible.

Second, the ability of the telemedical system to integrate real-time device and patient data using the IEEE 11073 SDC interoperability standard lays the groundwork for scalable, interoperable, and future-ready supervision models. As digital infrastructure in hospitals continues to evolve, systems like this could be enhanced with decision support tools, AI-based alerts, or predictive analytics to further assist both supervisors and residents.

Third, the study highlights the need for careful integration of new technology into clinical workflows. The increased temporal workload and the mixed feelings regarding remote observation underline the importance of proper training, user adaptation, and human-centered design. Future implementations should include training sessions as part of clinical education, possibly even integrating telemedical scenarios into simulation-based curricula.

Moving forward, real-world evaluations are essential to validate these findings in clinical practice. Pilot deployments in hospitals, supported by fallback mechanisms and mixed-modality supervision models, could test how the system performs under actual operating conditions. Additionally, future studies could expand the scope to include different critical events, surgical disciplines, or varying levels of clinician experience. Longitudinal research could also examine how repeated exposure and increasing familiarity affect both performance and user perceptions over time.

Finally, beyond anesthesiology, the architecture of such an interoperable telemedical system could be adapted for or combined with existing proprietary solutions for use in other high-acuity areas such as emergency departments, intensive care units, or interhospital consultations. With the global trend toward digital transformation in health care, establishing robust, interoperable TSV systems has the potential to reshape clinical collaboration and training, ultimately improving patient care and operational efficiency.

## Appendix

Multimedia Appendix 1 Translation and original document of the standard operating procedure for anaphylaxis at the Uniklinik Rheinisch-Westfälische Technische Hochschule Aachen.

## Abbreviations

AIXTRA: Aachener Interdisziplinäres Trainingszentrum für medizinische Ausbildung
EFRE: Europäischer Fonds für regionale Entwicklung
HPS: human patient simulator
IEEE: Institute of Electrical and Electronics Engineers
NASA: National Aeronautics and Space Administration
OR: operating room
PTZ: pan-tilt-zoom
RWTH: Rheinisch-Westfälische Technische Hochschule
SDC: Service-Oriented Device Connectivity
SOP: standard operating procedure
NASA-TLX: NASA Task Load Index
TSV: telesupervision
UI: user interface
