# Supplementary material for: Evaluating Telemedical Supervision for Critical Anesthesia Scenarios: Randomized Controlled Simulation Study
Source: JMIR Med Inform. 2026 Aug 18;14:e88056. doi: 10.2196/88056 (PMC13535306; doi:10.2196/88056)
Supplement: Supplementary file 1 [file medinform_v14i1e88056_app1.pdf]

Translation (German->English) of SOP for anaphylactic reaction at Uniklinik RWTH Aachen (original below)

Version: 1
Updated: 02-2018
Date: 1.12.2017

1. Objective and Purpose

Acute therapy and management of anaphylaxis in the perioperative setting and for the pain service.

2. Scope

Department of Anesthesiology at University Hospital Aachen, including acute pain service and pain clinic. For the emergency physician service, refer to the current version of the VAW "Anaphylaxis" of the Aachen city rescue service.

3. Description

3.1 Diagnosis:

Life-threatening ABC problems:

- A: Airway swelling, hoarseness, stridor
- B: Tachypnea, wheezing, fatigue, cyanosis, $SpO_2 < 92\%$, confusion
- C: Pallor, sweating, hypotension, weakness, drowsiness, unconsciousness
- **Intraoperative symptoms:**
  Skin symptoms, ventilation problems, SaO_2↓, CO_2↓, HR↑, BP↓, pulselessness
- **Note peri-/intraoperatively:**
  Patient unable to provide information, anesthesia-related BP fluctuations/blood loss, surgical drapes, multiple drugs via various routes in quick succession (gloves—e.g., latex—and disinfectants—e.g., chlorhexidine on indwelling catheters can also trigger)

Staging according to Ring and Messmer

Stage	Cardiovascular	Respiration	Skin	Other
I			Flush, urticaria, angioedema, itching	Malaise

Stage	Cardiovascular	Respiration	Skin	Other
II	Arrhythmias, hypotension	Rhinorrhea, hoarseness, dyspnea	Flush, urticaria, angioedema, itching	Nausea, cramps
III	Shock	Bronchospasm, laryngeal edema, cyanosis	Flush, urticaria, angioedema, itching (not required)	Vomiting, defecation, ↓consciousness
IV	Cardiac arrest	Respiratory arrest	Flush, urticaria, angioedema, itching (not required)	Vomiting, defecation, ↓consciousness

---

3.2 Therapy (ABCDE)

Basic Measures:

- Stop allergen exposure (if known)
- Inform senior physician/additional nursing staff
- Complete basic monitoring (possibly additional IV lines, arterial/Central venous catheter)
- Inform surgeon/possibly stop surgery
- Administer glucocorticoids (500–1000mg Solu-Decortin IV)
- Early H1-blockade:
  Dimetindene (=Fenistil®) 0.1 mg/kg (Clemastine (=Tavegil) 0.05 mg/kg). H2-blockade (Ranitidine 1mg/kg (50mg=1 vial)). Note: Little evidence for H2 blockade.
- Intensive care monitoring at least from stage III (note: 20% biphasic course approx. 6–24h after initial event)

A/B Airway/Breathing

- FiO2 = 100%
- Is the airway secure? Consider intubation (difficult airway possible!), emergency cricothyrotomy if feasible!
- Adrenaline nebulization
- β2-agonists inhaled (Salbutamol)
- β2-agonists IV (Reproterol)

C Circulation

- Volume substitution! (Crystalloids, no evidence for colloids)

- If necessary, vasopressors (norepinephrine, vasopressin)
- At the latest from stage III:
  Adrenaline IV (bolus 50 µg (ERC 2015)), titrate to effect. Alternatively, adrenaline IM in vastus lateralis 0.5mg
- If indicated, resuscitate as per ERC guidelines
- Consider glucagon in β-blocked patients if adrenaline is ineffective

D/E

- If GI involvement: suction, nasogastric tube, antiemetics, spasmolytics as needed
- For systemic skin manifestation & subjective symptoms:
  Basic measures (positioning, cooling, monitoring), consider H1 (& H2)-blockade

Medication dosing by age/weight:
(See table in original for details; e.g., Adrenaline IV bolus, adrenaline infusion, dimetindene, prednisolone, salbutamol, reproterol, volume bolus, oxygen by inhalation)

3.3 Premedication

Identify risk patients (e.g., with medication allergies):

- Allergic reactions in previous anesthesia
- Allergy card/certificate (legally binding)
- Patients with latex allergy or potential sensitization (e.g., spina bifida, multiple surgeries in infancy, medical personnel, cleaners, banana/avocado/latex cross-allergy)
- With contrast medium allergy, consider iodine/disinfectant!
- Children with multiple surgeries, spina bifida, frequent latex allergy/cross-allergy
- Patients with systemic mastocytosis
- No increased risk in atopic patients (allergic rhinitis, neurodermatitis, food allergy)

High risk for severe anaphylaxis (grade III–IV):

- Advanced age, serious heart disease, poorly controlled asthma, drugs promoting mast cell activation/leukotriene formation (e.g., NSAIDs, mastocytosis, β-blockers, ACE inhibitors)
- Premedication: 13h, 7h before: 50mg Decortin H orally, 1h before: 50mg Decortin H orally (if not possible: 100mg SDH IV), 5min before: H1 + H2 blocker
  (Dimentinden = Fenistil, Clemastin = Tavegil)

For systemic mastocytosis:

- These patients should be treated in coordination with the specialist clinic for systemic mastocytosis in Medical Department IV. Lab work should also be discussed there. (SOP in planning)

3.4 Prevention

- If latex sensitization suspected: use latex-free materials, schedule early in OR program, mark in OR plan
- Communicate with OR nurse and surgeon!
- Caution: contrast medium allergy and use of iodine/disinfectant, indocyanine green, amiodarone
- Avoid histamine-releasing substances if possible (e.g., thiopental, mivacurium)
- Administer substances slowly, in fractions, separately
- With suspected antibiotic allergy:
    - Medical History!
    - Surgeon must provide alternatives if penicillin/beta-lactam allergy
    - Consider administering antibiotics while patient is still awake so they can report symptoms

    Notes: Reaction can occur up to 6h after administration, even with test dose.
- Readiness to treat anaphylactic reactions if necessary

3.5 Diagnostic Confirmation

- Important **after** adequate acute care!
- Measure serum tryptase twice (via Medico order), plus IgE; specify all β-lactams if relevant
- Timing: earliest 1h, latest 3h after reaction, repeat at 24h. Serum tube can be stored at 4°C for 12h
- RAST lab and central lab contact info provided
- Inform patient, anesthesia card, summary of all drugs given in last 6h, info on materials used, detailed reaction description including grading, therapeutic measures, and course
- Recommendation for allergy work-up, preferably within 1 year

---

3.6 Follow-up care:

Not covered in the study

---

4. Documentation

- Document relevant allergies on first page of anesthesia record and in special fields; tick latex allergy
- Document allergic reaction in anesthesia journal
- For severe reactions with resuscitation, consider an incident report and CIRS report; document diagnostic measures and dermatology consult

---

5. Referenced Documents

- SOP infection control and prevention
- SOP resuscitation

---

6. History of Changes

Rev.	Date	Description of Change
00	08.02.2018	a) Table on p.7 extended with “Circulation” b) 3.5 updated and specified

---

Prepared by Gillmann, Langen; Checked and Released

Klinik für Anästhesiologie

Titel Anaphylaktische Reaktion 01

Version: 1

aktualisiert am 02-2018 Datum 1.12.2017

1 Ziel und Zweck

Akuttherapie und Management der Anaphylaxie im perioperativen Umfeld und im Schmerzdienst.

2 Geltungsbereich

Klinik für Anästhesiologie im Universitätsklinikum Aachen incl. Akutschmerzdienst und Schmerzambulanz. Für den Notarztdienst wird auf die VAW „Anaphylaxie" des Rettungsdiensts der Stadt Aachen in der aktuellen Fassung verwiesen.

3 Beschreibung

Therapie nächste Seite !

3.1 Diagnosestellung:

Lebensbedrohliche ABC Probleme:

- A: Schwellung der Luftwege, Heiserkeit, Stridor
- B: Tachypnoe, Giemen, Müdigkeit, Zyanose, $SpO_2 < 92\%$, Verwirrtheit
- C: Blässe, Schwitzen, Hypotonie, Schwäche, Schläfrigkeit, Bewusstlosigkeit
- **Intraoperativ Symptome**:
  Hautsymptome, Beatmungsprobleme > $SaO_2\downarrow$, $CO_2\downarrow$, HF↑, RR↓, Pulslosigkeit
- **Cave peri-/intraoperativ:** Pat. nicht auskunftsfähig, narkosebedingte RR-Schwankungen/Blutverluste, chirurgische Abdeckung, multiple Medikamente über versch. Applikationswege in kurzen zeitlichen Abständen (auch OP-Handschuhe - z.B. Latex und Desinfektionsmittel - z.B. Chlorhexidin auf Dauerkatheter können Auslöser sein),

Stadieneinteilung nach Ring und Messmer

Stadium	Herz-Kreislauf	Respiration	Haut	sonstiges
I			Flush, Urtikaria, Angioödeme, Juckreiz	Unwohlsein
**II**	Arrhythmien Hypotonie	Rhinorhoe, Heiserkeit, Dyspnoe	Flush, Urtikaria, Angioödeme, Juckreiz	Übelkeit, Krämpfe
**III**	Schock	Bronchospasmus, Larynxödem, Zyanose	Flush, Urtikaria, Angioödeme, Juckreiz (nicht obligat)	Erbrechen, Defäkation, Bewusstsein ↓
**IV**	Herz-Kreislauf-Stillstand	Atemstillstand	Flush, Urtikaria, Angioödeme, Juckreiz (nicht obligat)	Erbrechen, Defäkation, Bewusstsein ↓

© KQRM, nur für den UKA-internen Gebrauch bestimmt, Ausdrucke unterliegen nicht dem Aktualisierungsservice

3.2 Therapie (ABCDE)

Therapie

Basismaßnahmen:

- Allergenzufuhr stoppen (sofern bekannt)
- Fach-/Oberarzt und zusätzliche Pflege
- Basismonitoring vervollständigen (ggf. weitere PVK, Art/ZVK)
- Operateur informieren/ggf. Operations-Stopp
- Glukokortikoide (500-1000mg Solu-Decortin i.v.)
- Frühzeitig H_1-Blockade: CAVE Anticholinerge UAW
  Dimentinden (=Fenistil®) 0,1mg/kg (Clemastin (=Tavegil) 0,05mg/kg)
  H_2 Blockade (Ranitidin 1mg/KG (50mg = 1 Ampulle)Bem: Es gibt wenig Evidenz für die H_2 Blockade.
- Indikation zur Intensivüberwachung spätestens ab Stadium III
  (CAVE 20% mehrphasige Verläufe a.e. 6-24h nach Erstereignis)

A/B Atemweg/Beatmung

- FiO_2 = 100%
- Atemweg gesichert? Ggf. ITN CAVE: Difficult Airway; Notfallkoniotomie im Rahmen des Möglichen!
- Adrenalin-Verneblung
- ß$_2$-Sympathomimetika inhalativ (Salbutamol)
- ß$_2$-Sympathomimetika i.v. (Reproterol)

C Kreislauf

- Volumensubstitution! (Kristalloide, keine Evidenz für Kolloide)
- Ggf. Vasopressoren (NA, Vasopressin)
- Spätestens ab Stadium III :
  Adrenalin i.v. (Boli 50 µg (ERC 2015))– Titration nach Wirkung
  alt. Adrenalin (i.m. ► M. vastus lat 0,5mg)
- Wenn Indiziert Reanimation nach ERC-Guidelines
- Erwäge Glucagon bei ß-blockierten Patienten, falls Adrenalin keine Wirkung zeigt
  (zu finden im Notfalldepot, NOTA)

D/E

- bei gastrointestinaler Beteiligung: Absaugung, Magensonde, ggf. Antiemetika, Spasmolytica
- systemisch generalisierte Hautmanifestation u. subjektive. Symptome:
  Basismaßnahmen (Lagerung, Kühlung, Überwachung)
  H_1(&H_2)-Blockade erwägen
- **Pharmakotherapie für Kinder, Jugendliche und Erwachsene unter Intensivbedingungen**
  (nach S2 Leitlinie) [modifiziert für Gebrauch im OP]

Wirkstoff	Applikationsweg	< 15 kg KG	15–30 kg KG	> 30–60 kg KG	> 60 kg KG
**Adrenalin**	i.v. Bolus[1]	10µg/kg KG	10µ/kg KG	5-10µg/kg KG	5-10µg/kg KG
**Adrenalin**	Perfusor	0,05–1,0 µg/kg/min	0,05–1,0 µg/kg/min	0,05–1,0 µg/kg/min	0,05–1,0 µg/kg/min
**Adrenalin**	per Vernebler	2 ml[2]	2 ml[2]	2 ml[2]	2 ml[2]
**Dimetinden**	intravenös	1 ml[3]	2–3 ml[3]	4 ml[3]	8 ml[3] oder 1 ml/10 kg KG
**Prednisolon**	intravenös	50 mg	100 mg	250 mg	250–1.000 mg
**Salbutamol,**	per Vernebler	2 Amp=2,5 mg	2 Amp=2,5 mg	2 Amp=2,5 mg	2 Amp = 2,5 mg
**Reproterol[4]**	Dauerinfusion	0,1 µg/kg/min	0,1 µg/kg/min	0,1 µg/kg/min	0,1 µg/kg/min
**Volumen**	Bolus	20 ml/kg KG	20 ml/kg KG	10–20ml/kgKG	10–20 ml/kg KG
**Volumen**	Infusion (balanciertes Kristalloid)	1–2 ml/kg/min	1–2 ml/kg/min	1–2 ml/kg/min	1–2 ml/kg/min
**Sauerstoff**	inhalativ	2–10 l/min	5–12 l/min	5–12 l/min	5–12 l/min

[1] Für die Bolusgabe wird von einer 1 mg/ml Adrenalinlösung 1 ml mit 9 ml NaCl 0,9 % verdünnt (Endkonzentration 100 µg/ml); [2]für die Inhalation wird die Stammkonzentration verwendet (1 mg/ml); [3]einer (Stamm-) Konzentration von 1 mg/ml (1 ml = 1 mg); [4]Reproterol kann auch als Bolus gegeben werden

3.3 Prämedikation

Risikopatienten identifizieren - Pat. mit Medikamentenallergien

- Klinische Zeichen einer allergischen Reaktion in vorausgegangener Anästhesie.
- Allergiepass, Narkoseausweis, Attest (Bem.: sind juristisch bindend)
- Pat. mit Latexallergie, bzw. möglicher Latexsensibilisierung wie Patienten mit Spina bifida, Mehrfachoperationen im Kleinkindalter, Patienten aus dem medizinischen Bereich und Reinigungskräfte, Unverträglichkeit von Bananen, Avocado, Lokalreaktionen bei Verwendung von Kondomen, Luftballons.
- Bei Allergien gegen KM auch an Jod und Desinfektionsmittel denken!
- Kinder mit multiplen Operationen, Spina bifida. häufige Latexallergie bzw. Kreuzallergie (=Reaktionen auf Avocado, Banane, Feige, Mango, Papaya)
- Pat. mit systemischer Mastozytose (CAVE z.B. system. Mastozytose im Rahmen einer Mastzellleukämie, Urticaria pigmentosa)
- KEIN erhöhtes Risiko bei Atopikern (allerg. Rhinitis, Neurodermitis, Nahrungsmittelallergie,)
- In der neuen Medicoversion gibt es oben links eine kleine rote Raute mit einem „A“ als Allergiemarkierung. Wenn man mit der Maus draufhält, bekommt man weitere Informationen.

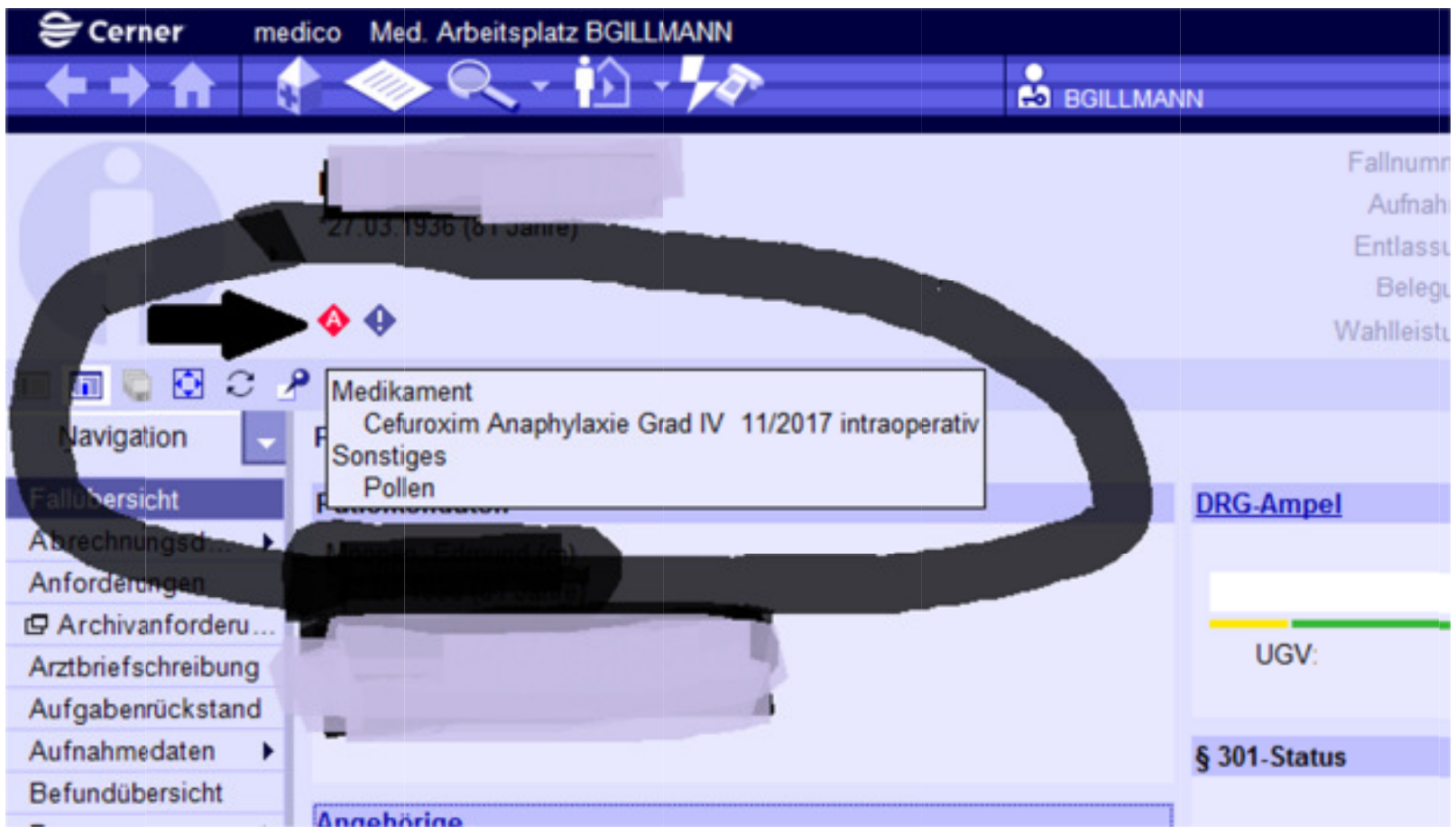

Risiko für schwere anaphylaktische Reaktion (Grad III-IV)

- Hohes Lebensalter; schwere Herz-Kreislauferkrankung, <u>schlecht eingestelltes</u> Asthma bronchiale, Einnahme von Medikamenten, die Mastzellaktivierung/Leukotrienbildung fördern z.B. NSAR, Mastozytose, Medikation mit ß-Blockern oder ACE-Hemmern

- Empfehlung Prämedikation Dermatologie UK Aachen (Quelle Dermatologie)für Risikopatienten mit schwerer vorhergegangener Reaktion (Grad III) falls Auslöser nicht sicher vermieden werden können:.
- 13h, 7h 50mg Decortin H oral, 1h präoperativ 50mg Decortin H p.o.
  (wenn p.o. Applikation nicht zeitgerecht erfolgt 100 mg SDH i.v.)
- 5 min präoperativ H1- und H2 Blocker
- *H1: Dimentinden i.v. (=Fenistil® 0,1mg/kg max. 4 mg i.v.) (Clemastin (=Tavegil®) 0,05mg/kg max 2 mg i.v.)*

Bei systemischer Mastozytose

- Diese Patienten sollten in Abstimmung mit der Spezialsprechstunde für systemische Mastozytose in der Med. IV behandelt werden. Dort auch Labor besprechen. (SOP in Planung)

3.4 Prävention:

- Bei V.a. Latexsensibilisierung: Latexfreie Materialen, früh im OP-Programm, Markierung im OP Plan.
- Nochmals deutlich mit OP-Pflege und Operateur kommunizieren!
- Cave bei „Kontrastmittelallergie“: Abwaschen mit jodhaltigen Desinfektionsmittel und Applikation von Indocyanin-Grün (PCH, NCH, AU) und Amiodaron
- Histamin-freisetzende Substanzen meiden (sofern das bekannt ist)(z.B. Thiopental, Mivacurium)
- Substanzen langsam, fraktioniert, separat applizieren
- Bei V.a. Antibiotika-Allergie (z.B. frgl. Penicillin-Allergie):
    - Anamnese!
    - Operateur muss Alternative (andere Wirkstoffgruppe z.B. Betalaktamantibiotikaallergie (Keine Penicilline, Aminopenicilline, Cephalosporine, sofern keine gesicherte Ausweichtestung vorliegt) alternativ gem. „SOP Infektionskontrolle und Prävention“ benennen oder Empfehlungen Hygiene auf der „Antibiotikakarte
    - Erwäge: Antibiotikagabe, wenn Patient noch wach ist u. über Symptome berichten kann.
      Bemerkung: Genauso wie bei einer Testdosis muss man sich aber bewusst sein, dass eine Reaktion bis zu 6 h nach Applikation stattfinden kann.
- Bereitschaft ggf. anaphylaktische Reaktion zu therapieren

3.5 Diagnosesicherung

- Wichtig **nach**! adäquater Akutversorgung
    - 2 x Serumtryptasebestimmung Anmeldung über Medico > Anforderung > ACAC/CAP Labor > Tryptase + IgE
    - Ggf. bei „Spezifische Untersuchungen“ weiter differenzieren. Bei ß-Lactamen immer alle aus der Gruppe ankreuzen
        - <u>Entnahmezeitpunkte:</u>
            - frühstens 1 spätestens 3 h nach Anaphylaxiereaktion
            - Wiederholung nach 24 h
    - 1 Serumröhrchen (braun oder weiß) Blutröhrchen kann 12h bei 4° gekühlt werden! (Eisbecher)
    - Das RAST Labor (Etage 5, Flur 12 Raum 8) ist nur tagsüber für den Läufer erreichbar. Sonst im Zentrallabor mit Notiz gekühlt zwischenlagern lassen. Telefonisch ankündigen.
    - Telefon: RAST Labor 89732 Zentrallabor 88066. Dermatologe v.D: via Zentrale (nicht Nachts im Haus)

© [Organisationseinheit], nur für den UKA-internen Gebrauch bestimmt, Ausdrucke unterliegen nicht dem Aktualisierungsservice

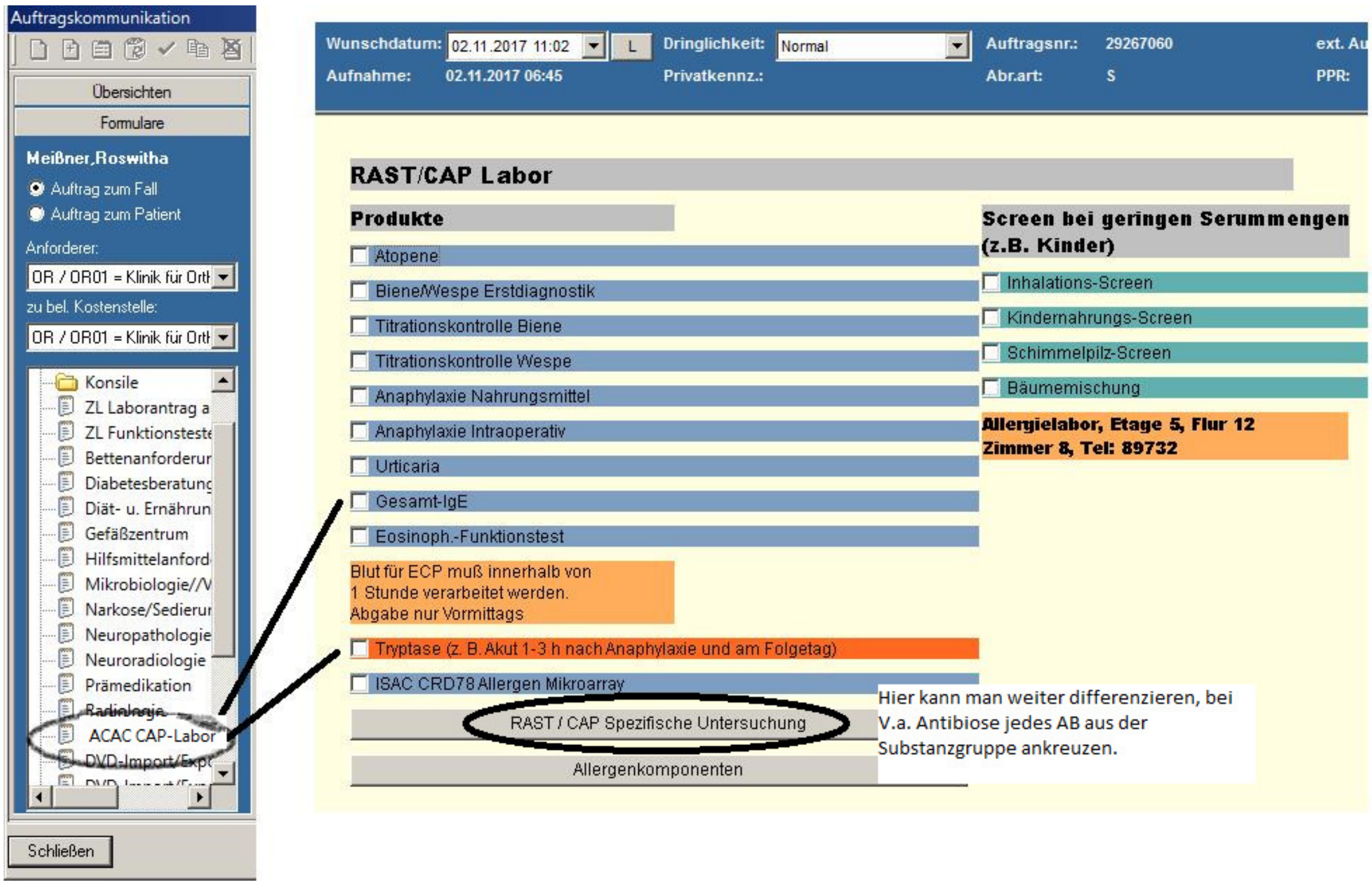

Ergebnisse des RAST Labors finden sich folgendermaßen:

Medico alt

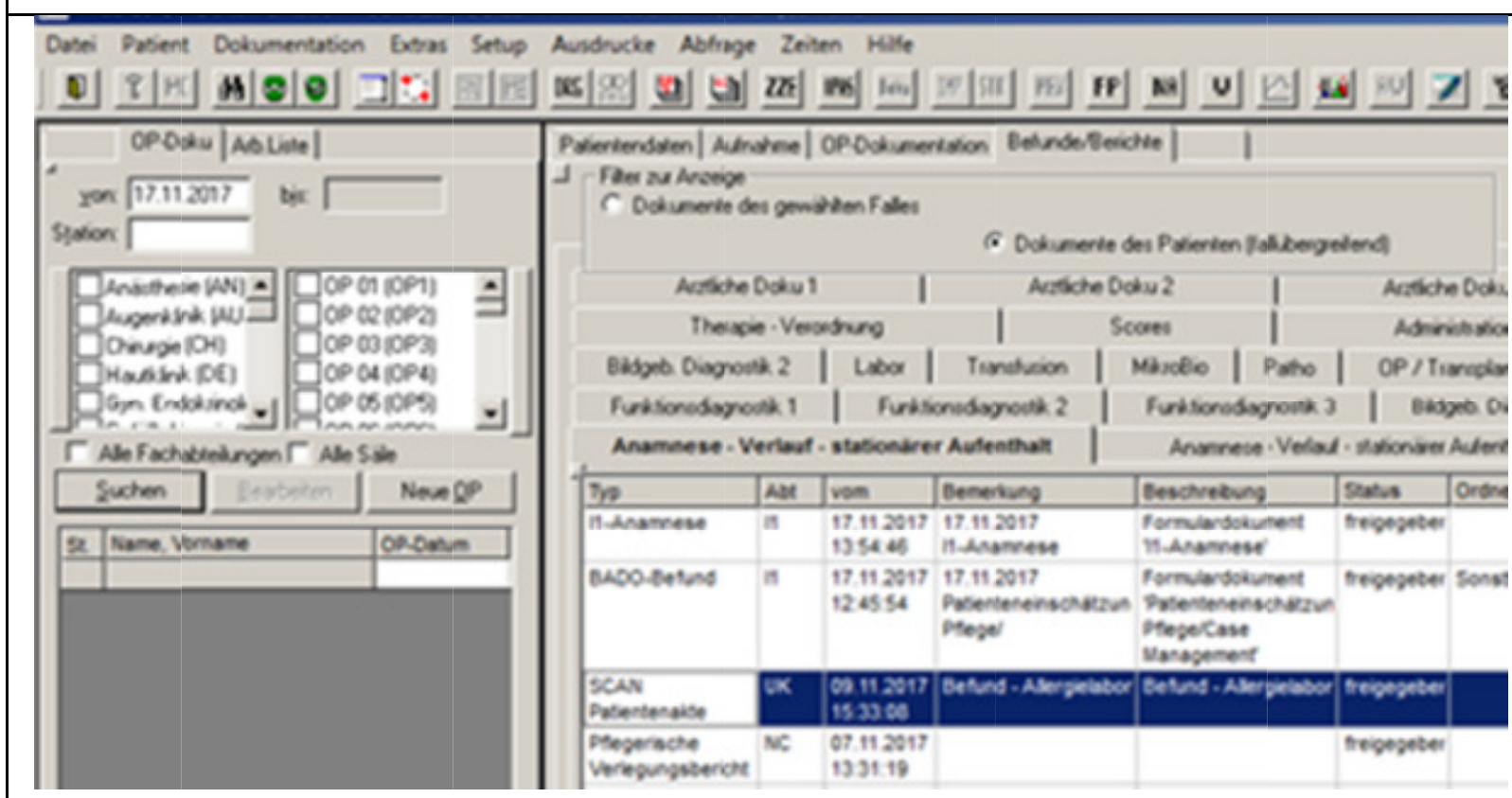

Medico neu

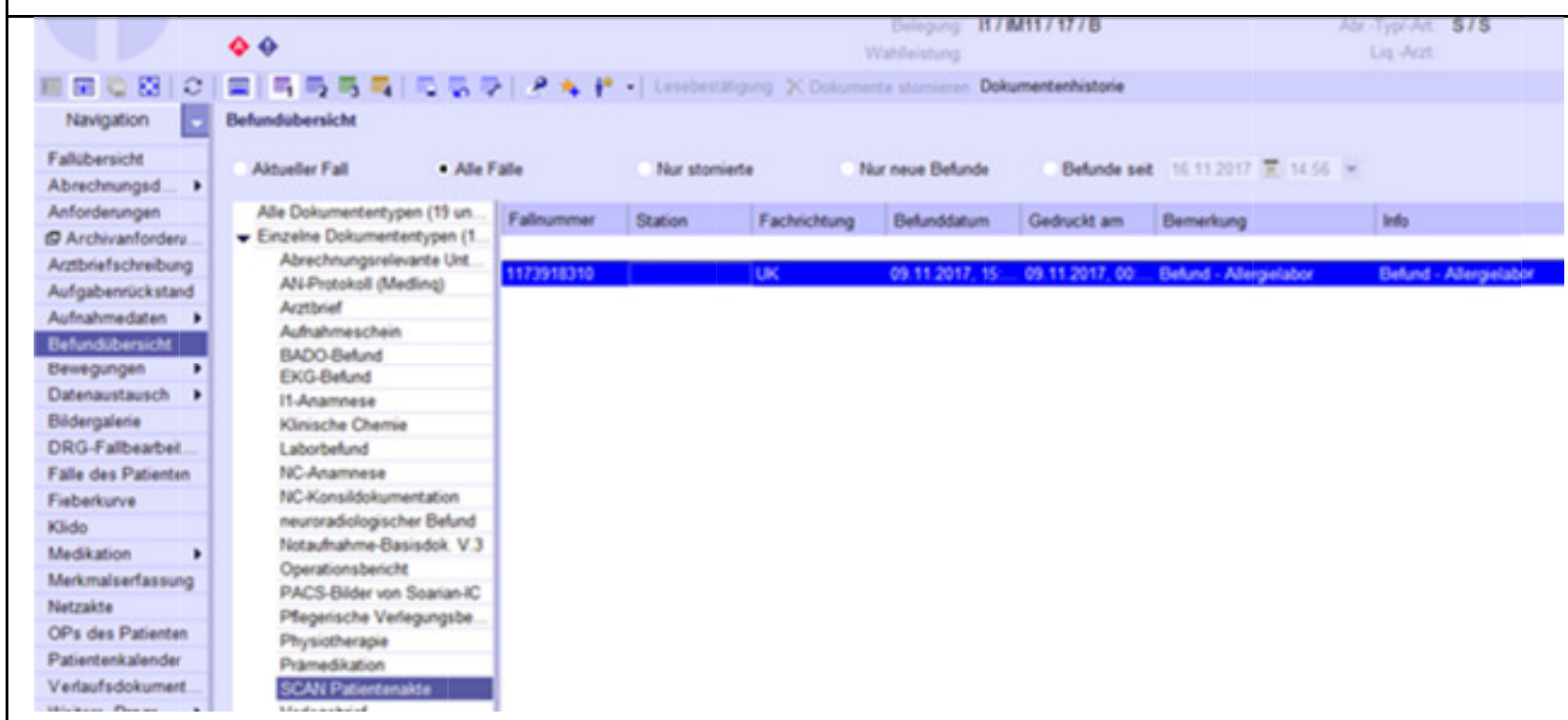

3.6 Nachsorge:

- Pat. informieren
- Narkoseausweis
- Zusammenfassung aller verabreichten Medikamente bis zu 6 h vor Anaphylaxie (Prämedikation, Eigenmedikation usw.), Info über periop. verwendete Materialen (Handschuhe, Desinfektionsmittel, Gleitmittel, Kontrastmittel), detailllierte Beschreibung der anaphylaktischen Reaktion mit Schweregradeinteilung, Therapiemaßnahmen und Verlauf). *Vordruck am Ende der SOP.*
- Empfehlung zur Allergologischen Abklärung vorzugsweise innerhalb 1 Jahres, da im Verlauf die Nachweisbarkeit deutlich abnimmt.
- Ggf. Eintrag einer Allergie im Medico (neu)
  Merkmalserfassung > Merkmaltyp „Allergie“, dann Merkmalsart „Medikament“ und weitere Informationen in das Freitextfeld „Zusatzinformationen“. (Soll in ein strukturiertes Feld geändert werden)

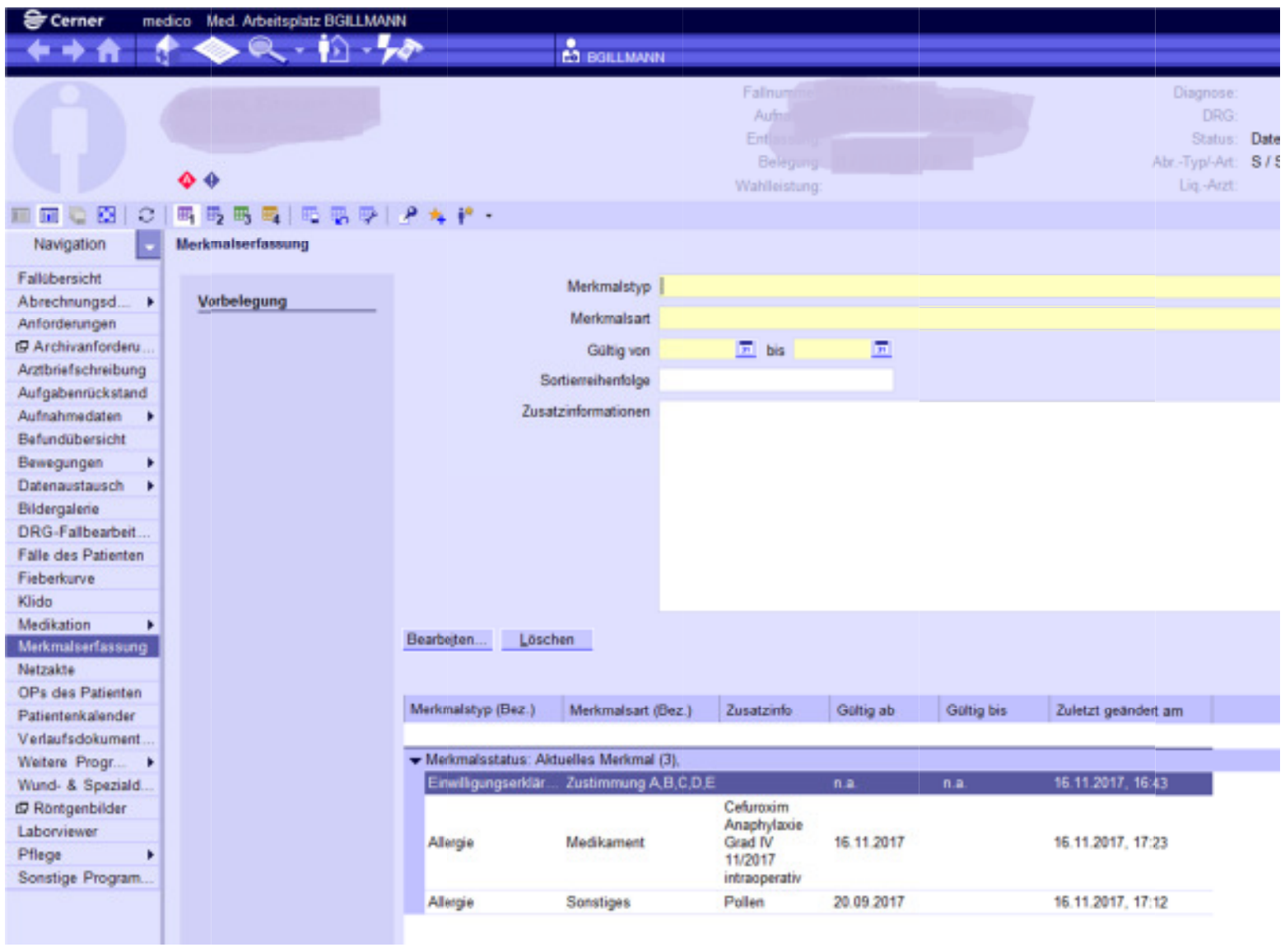

Klinik für Anästhesiologie
Dokumentationsbogen zur Diagnosesicherung bei Verdacht auf intraoperative Anaphylaxie

Patientenaufkleber

Datum der Narkose
Anaphylaktische Reaktion Grad :
Führendes Symptom: Kreislauf Pulmonal Kutan

Folgende Medikamente sind bei der perioperativen allergischen Reaktion im Rahmen der Narkose appliziert worden. Es werden die jeweils aktuellen Generika/Produkte des UKA verwendet.

**Opiate**	Fentanyl	Remifentanil	Sufentanil	Piritramid	Morphin
**Hypnotika**	Propofol 1%	Propofol 0,5%	Thiopental	Etomidate	Midazolam
**Muskelrelaxantien**	Succinylcholin	Rocuronium	Atracurium	Mivacurium	Vecuronium
**Periphere Analge-tika**	Metamizol	Dynastat® = Parecoxib	Paracetamol	Ibuprofen	Diclofenac
**Infusionen**	Ringer	Gelafundin	NaHCO3	Paed G1	Mannit-Lsg 15%
**Sonstige**	S-Ketamin	Clonidin	Atropin	Ondansetron	
**Kreislauf**	Norepinephrin	Epinephrin	Akrinor	Urapidil	
**Antibiotika**	Cefuroxim	Clindamycin	Ampicillin/ Sul-bactam	Metronidazol	Piperacillin/ Tazobactam
**Lokalanästhesie**	Lidocain	Ropivacain	Bupivacain	Prilocain	Mepivacain
**Kontaktstoffe**	Octeniderm	Handschuhe	Pflaster	Sonogel	Instellagel
**Katheter**	RA-peripher	PDK	ZVK	Art	Veneverweil

Sonstige Medikamente/Erläuterungen:

Eigenmedikation:

Verdächtige Substanz:
Ggf. PZN und CH Nummer:

Anästhesist/in (Name, DECT):
Oberärztin/arzt (Name, DECT):

☑ Tryptase / IgE abnehmen ☑ Fotokopie des Narkosejournals

itte diese Seite ausdrucken

3.7 Literatur

1. Ring, J., et al., Guideline for acute therapy and management of anaphylaxis: S2 Guideline Allergo J Int, 2014. 23(3): p. 96-112.
2. Kannan, J.A. and J.A. Bernstein, Perioperative anaphylaxis: diagnosis, evaluation, and management. Immunol Allergy Clin North Am, 2015. 35(2): p. 321-34.
3. Niebel, P. and H. Wulf, [Declaration of Helsinki on patient safety in anesthesiology--part 4: SOP for perioperative anaphylaxis]. Anasthesiol Intensivmed Notfallmed Schmerzther, 2013. 48(4): p. 230-2.
4. Michalska-Krzanowska, G., Tryptase in diagnosing adverse suspected anaphylactic reaction. Adv Clin Exp Med, 2012. 21(3): p. 403-8.
5. Mertes, P.M., et al., Reducing the risk of anaphylaxis during anesthesia: 2011 updated guidelines for clinical practice. J Investig Allergol Clin Immunol, 2011. 21(6): p. 442-53.
6. Berrio Valencia, M.I., Perioperative anaphylaxis. Braz J Anesthesiol, 2015. 65(4): p. 292-7.
7. Allergo J Int 2014; 23: 100 Leitlinie zu Akuttherapie und Management der Anaphylaxie S2-Leitlinie
8. Notfall + Rettungsmedizin December 2015, Volume 18, Issue 8, pp 833–903 Kapitel 4 der Leitlinien zur Reanimation 2015 des European Resuscitation Council

4 Dokumentation

Auf der ersten Seite des Narkosejournals werden relevante Allergien im entsprechenden Feld dokumentiert. Eintrag in das Freitext Feld „Besonderheiten“ bzw. „Latexallergie“ ankreuzen in der Medico Prämedikations-maske.

Narkosejournal: Allergische Reaktion dokumentieren. AVB angekreuzen.
Bei schweren allergischen Reaktionen mit medikamentöser und/oder mechanischer Reanimation ist ein Zwischenfallsbericht zu erwägen, ggf. CIRS Report. Maßnahmen zur Diagnosesicherung wie Laborentnahme und Initiierung eines dermatologischen Konsils sind ebenso zu dokumentieren.
Davon unberührt bleibt ein Eintrag in den Narkoseausweis.

5 Mitgeltende Unterlagen

SOP Infektionskontrolle und Prävention

SOP Reanimation

6 Änderungsnachweis

Rev.	1. Datum	2. Beschreibung der Änderungen
00	08.02.2018	a) Tabelle S. 7 um „Kreislauf“ ergänzt b) 3.5 aktualisiert und spezifiziert

Datum

Erstellt Gillmann, Langen | Prüfung | Freigabe

© [Organisationseinheit], nur für den UKA-internen Gebrauch bestimmt, Ausdrucke unterliegen nicht dem Aktualisierungsservice
